# Liuzijue Qigong vs traditional breathing training for patients with post-stroke dysarthria complicated with abnormal respiratory control: study protocol of a single center randomized controlled trial

**DOI:** 10.1186/s13063-018-2734-0

**Published:** 2018-06-26

**Authors:** Hongli Li, Gaiyan Li, Gongliang Liu, Ying Zhang

**Affiliations:** Department of Rehabilitation, the Central Hospital of Xuhui District, No. 966 Middle Huaihai Road, Xuhui District, Shanghai, 200031 People’s Republic of China

**Keywords:** Dysarthria, Post-stroke, Abnormal breathing control, Six character formula, *Liuzijue qigong* (*LQG*)

## Abstract

**Background:**

Stroke-induced dysarthria is caused by muscle weakness, sacral or muscular dystonia, and incoordination of the articulatory organ formed by organic lesions caused by cerebral vascular obstruction or sudden bursting of blood vessels in the brain, which may cause abnormal breathing patterns, pronunciation, resonance, rhythm, and unclear articulation.

The Six Character Formula, or *Liuzijue qigong* (*LQG*), is an essential part of Chinese traditional exercises and focuses on breathing–speech synchronization. The purpose of the present study was to compare the effects of *LQG* with traditional breathing training (combined with basic articulation training in both groups) in patients with post-stroke dysarthria.

**Methods/design:**

The proposed study will be a single-center randomized controlled trial. A total of 100 patients, with a modified Frenchay Dysarthria Assessment (FDA) dysarthria assessment score < 27 and with a FDA speech breathing level ≥ b will be randomly divided into study (*LQG*, *n* = 50) and control (conventional breathing training, n = 50) groups. Basic articulation training will be conducted once a day, five times a week for 3 weeks. Data collection will be conducted at baseline, 1 week, and 2 weeks post-treatment initiation and after completion of the treatment (3 weeks). Comprehensive analyses will be conducted to measure and compare any differences in speech breathing dysfunction levels, comprehensive evaluation of dysarthria, maximum phonation time (MPT), maximal counting ability, signal-noise (S/Z) ratio, and loudness scales between the study and control groups.

**Discussion:**

This trial will provide evidence about the effectiveness of *LQG* for improvement of speech breathing function and speech ability in patients with post-stroke dysarthria complicated with abnormal breathing.

**Trial registration:**

Chinese Clinical Trial Registry, ChiCTR-INR-16010215. Registered 21 December 2016.

**Electronic supplementary material:**

The online version of this article (10.1186/s13063-018-2734-0) contains supplementary material, which is available to authorized users.

## Background

Dysarthria is a motor speech disorder caused by weakness, paralysis, or a lack of coordination of the motor-speech system [1]. It has been estimated that in the US 795,000 people will experience a new or recurrent stroke in 2013 [2] and 36–57% of individuals will have post-stroke dysarthria [3], which is often characterized by slurred, slow, breathless, or imprecise speech [4]. Individuals with post-stroke dysarthria often have problems forming understandable conversations [5] leading to emotional problems with social participation [6]. The recovery of post-stroke dysarthria is dependent on the improvement of breath control ability since the basis of sound is supported by the volume and control of respiratory airflow [7]. Intensive voice treatment, such as Lee Silverman voice treatment (LSVT) [8], has shown improved articulation, facial expression, and swallowing in patients with Parkinson’s disease (PD) [9] and some positive effects in other neurological disorders (stroke and cerebral palsy) [10]. Although non-speech oro-motor exercises (NSOMExs) are used in speech and language therapies for neurological disorders, especially stroke and other dysarthria classes, results on speech outcomes affected by NSOMExs have been inconclusive [11, 12]. The fact that post-stroke individuals often experience dysarthria complicated with abnormal breath control highlights the need to form a proper and efficient pattern of coordination between breath and speech, which is more likely to benefit patient coordination and help restoration of normal speech [13]. To date, traditional breathing training programs, such as blowing paper, candles, and ink and other exercises, deliberately force thoracic breathing into the abdominal breathing mode but ignore the synchronous treatment of pronunciation and breathing, blindly strengthening muscle strength, which makes the treatment program rigid.

The Six Character Formula, or *Liuzijue qigong* (*LQG*), is one of the New Health Exercise Series compiled by the China Qigong Management Center [14]. *LQG* has deep roots in traditional Chinese medicine and is a typical speech–breath therapy, which involves diaphragmatic breathing practice combined with the chanting of six different sounds (Xu, He, Hu, Si, Chui, Xi). The method is to inhale through the nose and then exhale through one of the six different tongue and mouth shapes, and also comply with appropriate body movements and mind-control breathing top-down or vice versa.

Recent studies have shown that *LQG* promoted functional lung capacity and improved the quality of life in older adults with chronic obstructive pulmonary disease (COPD) [15] and also influenced heart rate variability (HRV) in different postures [16]. It has also been demonstrated that a Health QiGong program, in which Xu and Xi exercises from *LQG* were included, could reduce the symptoms of PD and improve the body functions of PD patients [17]. Since *LQG* synchronizes breathing and speech and increases respiratory muscle strength and coordination, we hypothesize that it might help patients with post-stroke dysarthria, complicated by abnormal breathing control, to improve stability and the utilization efficiency of flow to establish normal breathing patterns for speech. Therefore, the aim of this study will be to investigate the therapeutic effect of the Six Character Formula in patients with post-stroke dysarthria complicated with abnormal respiratory control compared with traditional breathing training.

## Methods and design

### Trial design

**Fig. 1:**
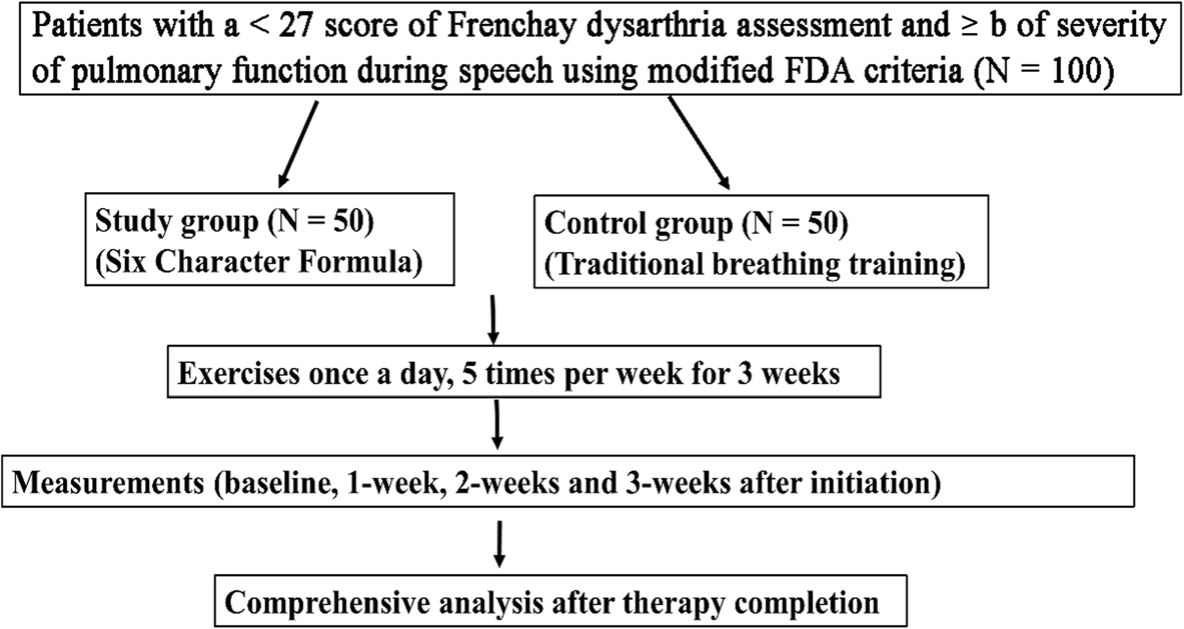
Flow diagram of the study. Pulmonary function during speech will be tested and graded according to the FDA [25]: a--No difficulty; b--Inhalation or exhalation not smooth or is shallow; c--Marked interruptions of inhalation or exhalation, or difficulty of inhaling deeply; d--little control rate of inspiration or expiration- short of breath may appear. More consistently impaired than c; e--Patient unable to attempt task. No control

**Fig. 2:**
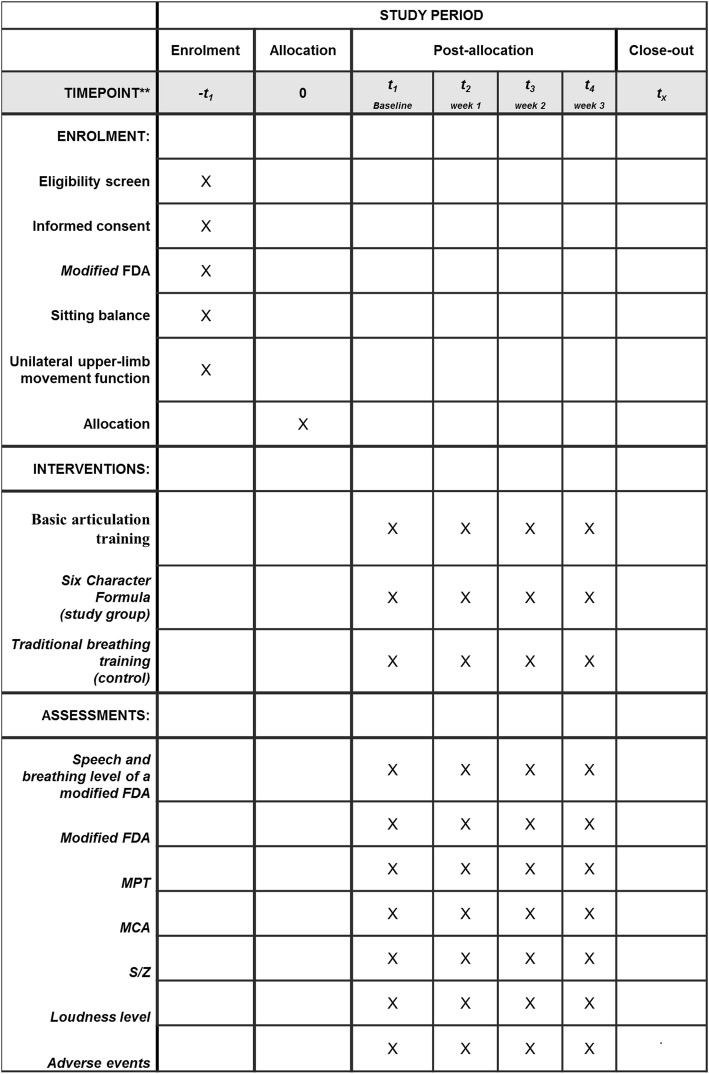
SPIRIT figure of the Liuzijue Qigong study protocol

Our proposed study is a single-center randomized controlled trial comparing the therapeutic effect of two breathing training methods (Six Character Formula vs traditional breathing training) in patients with post-stroke dysarthria complicated with abnormal respiratory control. The study design is depicted in the flow diagram shown in Fig. 1. The research protocol has been designed according to the SPIRIT recommendations (Fig. 2 and Additional File 1)

### Primary and secondary objectives of the study

In this study, we selected the “speech breathing level of a modified Frenchay Dysarthria Assessment (FDA)” as the primary objective indicator. At the same time, the curative effect will be assessed as complete response (CR), partial response (PR), and no response (NR) to assess statistically the effectiveness before and after treatment. The indicator will be ranked data, and the statistical method to be adopted is shown in Additional file 2: Table S1.

The study will also select modified FDA, maximum phonation time (MPT), maximal counting ability (MCA), signal-to-noise (S/Z ratio), and loudness level as secondary objective indicators. The data obtained from these indicators will be measurement data and data from the loudness level will be ranked data.

### Primary outcome measurement procedures

Outcome data will be collected at baseline, 1 week, and 2 weeks after the initiation of treatment, and also after the completion of the treatment (third week), by experienced physicians.

The primary measurement will be the speech breathing level of the modified FDA (Table 1).

**Table 1:** Specific measurement of speech breathing level assessed by modified FDA

	Baseline	1 week	2 weeks	3 weeks
Speech breathing specific measurement				
Evaluation date				
Signature of therapist				

Speech breathing will be evaluated by letting the patients count to 20 as fast as possible in one breath. They will be assured not to be concerned about the articulation and just the number of breaths necessary to complete the task will be monitored. The normal outcome for this task is the performance in one breath.

The level of speech breathing-specific measurements will be defined as: a) normal and the number of breaths used as the baseline to quantify the seriousness of speech breathing dysfunction; b) the patient needs one additional deep-breath to smooth the speech; c) patient needs four deep breaths due to the disappearance of voice; d) patient needs seven deep breaths due to obvious variability of speech; e) patient spells one word with one breath due to serious impairment of speech. Subjects will be given a score of 1 for grade a, 2 for grade b, 3 for grade c, 4 for grade d, and 5 for grade e. The primary indicator will be the breath assessment of FDA.

The curative effect will be assessed as: 1) CR if the original level degrades ≥ 2 or reduces to the level of grade a; 2) PR if the original level degrades by 1; and 3) NR if the original level does not change or increases.

### Secondary outcome measurement procedures

The secondary outcome measurements will include the comprehensive evaluation of dysarthria using modified FDA, MPT, MCA, S/Z ratio, and a loudness scale (Table 2) at baseline, 1 week, and 2 weeks post-treatment initiation and after completion of treatment (third week) by experienced physicians.

**Table 2:** Comprehensive evaluation of dysarthria using modified FDA, maximal counting ability, signal-to-noise (S/Z) ratio and loudness scale

	Baseline	1 week	2 weeks	3 weeks
Comprehensive evaluation of dysarthria				
Maximum phonation time (s)				
Maximal counting ability (s)				
S/Z ratio				
Loudness scale				
Evaluation date				
Signature of therapist				

The comprehensive evaluation of dysarthria using a modified FDA is composed of eight major items (including reflex, respiration, lips, tongue, jaw, palate, larynx, and speech) and 28 sub-items. Each item and sub-item will be divided into five scales (from a to e) according to the level of impairment and “scale a” is used as the baseline to quantify the recovery of dysarthria; a higher “a” score means good recovery from dysarthria.

Maximum phonation time (MPT) will be measured for the longest time that a patient can pronounce the simple vowel *a* after deep breathing. Measurement of MPT should satisfy the following requirements: 1) duration of pronunciation as long as possible; 2) even breath flow; 3) even breath loudness; and 4) tone within correct frequency range. The relatively longer MPT value will be selected as the final outcome from two measurements, which meet the above-mentioned requirements (vide supra). The tone and loudness of pronunciation need to be kept at a comfortable level. The maximal counting ability (MCA) will be defined as the longest time that a patient can pronounce the letter “E” (one in Chinese) without a break after a deep inhalation. The S/Z ratio is the ratio of the longest time that a patient can pronounce *s* vs *z* after a deep breath in. The loudness scale will be determined by a therapist while talking with a patient and graded as whispered, soft, conversational, loud, or shouting.

### Adverse event collection procedure during the trial

The adverse events will be truthfully reported and handled and recorded in a timely manner. Follow-ups will be conducted to find out the reasons and to ensure the health, safety, and rights of the subjects. Serious adverse events will be reported within 24 h to the main researchers, the Clinical Trial Office of Xuhui Central Hospital, and the Ethics Committee of Shanghai Xuhui Central Hospital and the subjects will receive appropriate counseling.

### Trial setting

The study will be carried out in the Department of Rehabilitation Shanghai Xuhui Central Hospital. All patients will receive comprehensive rehabilitation provided by physiotherapists, including exercise therapy, occupational therapy, daily life ability training, and traditional physical therapy.

### Inclusion criteria

Subjects will have to satisfy all of the following criteria to be included in the study: 1) meet the diagnostic criteria of cerebral infarction or hemorrhage; 2) meet the traditional Chinese medicine (TCM) diagnostic criteria of apoplexy; 3) had their first brain stroke with dysarthria; 4) were diagnosed with a FDA score < 27 and dysarthria combined with a ≥ b scale of pulmonary function during speech according to the FDA criteria (revised by the Rehabilitation Center of Hebei Provincial People’s Hospital; 5) are in a phase 2 weeks to 6 months after stroke; 6) have enough physical strength to complete 40-min speech training; 7) have a sitting balance ranking ≥ 3; 8) have at least normal unilateral upper-limb movement function or Brunnstrom stage of ≥ 4; 9) have normal and stable vital signs; and 10) have a willingness and ability to provide written informed consent.

### Exclusion criteria

Subjects will not be enrolled if they meet any of the following exclusion criteria: 1) unconsciousness and severe cognitive impairment; 2) combined with aphasia; 3) incapable of completing 40-min speech breathing training; 4) have complications such as acute disease in major organs of the heart, brain, and kidney; 5) serious psychological disorders.

### Study population and recruitment

A total of 100 patients aged from 40 to 80 years who are diagnosed with stroke in the Department of Rehabilitation in Shanghai Xuhui Central Hospital from Dec 2016 to Dec 2019 will be recruited into the study.

The recruitment procedure of patients will involve four steps: 1) the patient’s attending physician will need to be familiar with the criteria for inclusion and exclusion of a patient into the study, screen potential subjects, and contact the primary researcher. 2) The researcher will introduce the trial to potential subjects and discuss the objectives with them based on the test requirements and schedule, and potential participants will be asked for their opinions. 3) According to the inclusion and exclusion criteria, the participants will be judged as eligible or ineligible for inclusion. 4) Participants need to sign a consent form to participate in the trial.

### Randomization

Subjects will be randomized 1:1 using simple randomization via random numbers generated in Excel. To make sure that the risk of bias remains low, patients will be registered in the database by means of a patient ID code so that assessors are blinded during the analysis. Only the primary investigator will have knowledge about the allocation. The outcome assessors and care providers will be different doctors. A special evaluation team will be set up and two senior speech therapists will undertake the assessment work. The outcome assessors and care providers will not exchange information during the implementation of the experiments, and the outcome assessors shall not ask the subject for the intervention to ensure accuracy of the assessment.

### Treatment

The *LQG* (study group) or traditional breathing (control group) training followed by basic articulation training (20 min for each) will be conducted five times per week for 3 weeks in a soundproof, 10 m^2^ room with a background noise of ≤ 30 dBA. Training will be provided by experienced investigators who will ensure that the process is performed in accordance with the protocol. Briefly, a patient will first need to adjust to a straight sitting position, perform relaxing breathing with help from regular upper-limb movement, and be instructed to breathe in smoothly through the nose and exhale slowly through the mouth.

### Control group

Patients allocated to the control group will start to breathe in for a count of 3 initiated by the therapist, hold their breath for another count of 3, and then exhale for 10 s from a count of 1. The therapist will also help to increase the patient’s expiratory flow by squeezing the area just above the costal arch on either side to exert pressure on the chest at the end stage of exhalation. The basic articulation training will include multidirectional movements of lips and tongue, the central part of the jaw, and the skin near the temporomandibular joint. Speech training will start from a single vowel (e.g., *a* or *u*) and consonant (e.g., *b*, *p*, *m*) to various combinations of vowel and consonant pronunciations, and gradually to single words and sentences.

### Study group

Patients assigned to the study group will receive Six Character Formula breathing training according to our improved scheme of the Healthcare Qigong Liuzijue instructions edited by the Healthcare Qigong Administration Center of State Physical Culture Administration. The method includes breathing in through the nose and exhaling with annunciation of one of the six different voices and breathing sounds (Xu, He, Hu, Si, Chui, and Xi). At the same time, each sound will be complemented by specific action guidance.

#### Oral guidance key points

Oral guidance key points are the key points of pronouncing and breathing of Liuzijue. For “Xu”, feel the upper and lower teeth (i.e., incisors) force with the two lips parted. For “He”, the strength originates from the root of the tongue, and the mouth is opened naturally. For “Hu”, the power of the voice is in the throat, and the mouth bulges forward like a tube. For “Si”, the power of the voice comes from the teeth when the two lips are slightly open, feeling the corners of the mouth move backwards. For “Chui”, the force is in the central part of the lips, and the middle of the two lips is slightly open. For “Xi”, the power of the voice comes from the upper oral cavity and throat—the two lips are slightly open and the incisors seem to be closed.

Before training, we will inform each patient of the “focus” when he/she breathes and pronounces. We will ask the patient gradually to feel the focus instead of requiring the patient to pronounce forcefully, so as to facilitate the accuracy of pronunciation and breathing.

#### Other key points

A patient with dysarthria after a stroke may have varying degrees of limb hemiplegia and abnormal posture and be unable to sit or stand. Therefore, it is often impossible to complete the action guidance of Liuzijue independently and accurately. Thus, the training position and guide actions cannot be made too rigid. Patients can take an alternative standing, sitting position, etc. A healthy upper limb can drive the affected upper extremity, or the therapist will stand next to the patient to help them complete the action to maintain the normal position of the thorax, spine, and pelvis and to provide a stable mechanical structure. With improvement in the patient’s ability to balance in the standing position, the training position will be gradually transitioned from the sitting position to an independent standing position. With improvement of the upper and trunk functions of the patient, the therapist will gradually reduce the amount of aid. Whether it is active or auxiliary, body movements are based on the principle of mild gentle restfulness. The goal will be to complete the accurate breath guidance protocol.

In addition, the therapist will measure the patient’s decibels before practicing the Six Character Formula and assist them to accomplish target decibel values with increased articulation and respiratory flow.

Basic articulation training will be conducted after training in both the study and control group, consisting of movement training (lips, tongue, and jaws) and pronunciation training from easy to difficult.

### Sample-size calculation

We anticipate that the effective rate of speech breathing-specific curative effect assessed by modified FDA will be 70 and 40% for the study and control groups, respectively. Under such an assumption, 41 participants per group (on a 1:1 ratio) will be needed to show a significant difference at a *P* value < 0.05 with a test power of 80%. Anticipating losses of 18% in follow-ups, we will establish a target sample size of 100 participants (50 participants in each group).

### Statistical analysis

In the study, the main indicator, “speech breathing level according to modified Frenchay Dysarthria Assessment (FDA)”, will be ranked data. The statistical method will be as follows. (1) The main indicator will be compared between baseline and follow-up time with a signed rank test. The score change will be compared between groups using a rank-sum test. Unless indicated otherwise, hypothesis testing will use a two-sided test with *P* = 0.05 as the statistical cutoff point. (2) The influence of rehabilitation intervention time, stroke classification, age, and gender on the efficacy of the intervention method and the relative size of the efficacy will be estimated using a stepwise logistic regression model. (3) The overall efficacy evaluation will be the effective rate. The formula will be: Effective rate = (Excellent + Effective)/Total number of cases × 100%. A chi-squared test will be used to compare any differences in efficacy between the two groups.

The secondary outcomes will be analyzed using a repeated measure covariance analysis with the outcome baseline adjusted for as a covariate. We will test whether there is a statistically significant difference between the groups using a parameter representing treatment allocation. The ‘loudness level’ outcome is ranked data and will be analyzed in a similar way to the primary outcome.

The per-protocol (PP) population will include the randomized patients of the intention to treat (ITT) population but exclude those for whom the inclusion or exclusion criteria were not met and those who did not receive the actual treatments to which the patients were randomly allocated [18]. If the statistical results of the ITT and PP population data are the same, the results will be deemed to be reliable; if they are contrary, we intend to adopt the results of the ITT population. The method of processing missing data will be the last observation carried forward (LOCF). This refers to assigning the last observed value of the end point indicator to the subsequent missing evaluation point; that is, the last observation response will be considered to be the study end point.

#### Trial quality control

The evaluation will be handled by a person in charge to establish unified evaluation and operation procedures.

#### Researcher training

Prior to the beginning of the project, the participating physicians will undergo unified training. Through training, the researchers will fully understand the purpose, plans, indicators, and the Case Report Form (CRF) filing of the clinical research program and will be required to adhere to the study concept. Each physician will have a “researcher’s manual” for easy reference.

## Discussion

Six Character Formula or *LQG* is one of the common forms of Chinese qigong focused on breath control through inhalation and exhalation of six sounds with a history of more than 1500 years. The exercises feature slow and gentle movement of the mouth and tongue, which is believed to regulate the rise and fall of qi (vital energy) through the meridian system in the body and to help nourish the corresponding organs [19, 20]. Since patients with dysarthria retain basic language ability but have muscular dysfunctions in controlling articulation, respiration, phonation, prosody, and resonation [21], we hypothesize that post-stroke dysarthria patients will overcome speech difficulties by practicing *LQG* to increase breathing control abilities, strengthening face and mouth musculature, and improving the movement of the mouth and tongue. We expect that *LQG* will result in gradual transition from costal breathing to abdominal breathing through guidance of limb movements and the combination of both articulation and mouth shape. Such breathing training is expected to be more acceptable by patients and will promote maximum recovery of their breathing coordination. Therefore, the current study is designed to compare the therapeutic effect of the Six Character Formula vs traditional breathing training in patients with post-stroke dysarthria complicated by abnormal breath control. There are six types of dysarthrias, including spastic, flaccid, ataxic, hyperkinetic, hypokinetic, and mixed [22–24], and each condition is characterized by a different etiology and different speech behaviors, as well as different levels of respiratory control abnormalities. FDA [25] is one of the formal assessment tools used to obtain a complete oral-facial examination and a method to identify the primary speech characteristics, which can contribute to the neurological diagnosis and treatment guidance. Patients with post-stroke dysarthria and simultaneous abnormal respiratory control will be selected as our study subjects through a modified FDA. Modified FDA and other examinations, including MCA, S/Z ratio, and loudness scale, will be conducted in the current study to comprehensively evaluate the therapeutic effects of the training therapies.

In summary, the proposed study will focus on dysarthria complicated by abnormal breathing control and intends to introduce *LQG* into post-stroke dysarthria rehabilitation by highlighting the breath–speech synchronous training exercise.

### Limitations of the study

The limitations of the study include the following: (1) due to the limited medical resources and the short hospitalization period, the intervention period for this study will only be 3 weeks; (2) the subjects will not be followed-up after intervention; (3) we cannot realistically achieve single-blind or double-blind conditions, which is a limitation of research on rehabilitation therapy; (4) the evaluation index is mainly subjective and objective—quantitative indicators will need to be added in future research.

### Trial status

#### Ongoing trial

We have not completed patient recruitment at the time of submission.

## Additional files

Additional file 1: SPIRIT 2013 Checklist. (DOCX 55 kb)

Additional file 2: **Table S1.** Primary and secondary objectives as well as their related statistical methods. The new table contains a short overview of indicators, used evaluation methods, and types of data and statistical methods for analyzing primary and secondary study objectives. (DOCX 15 kb)

## Abbreviations

COPD: Chronic obstructive pulmonary diseases
CR: Complete response
FDA: Frenchay Dysarthria Assessment
HRV: Heart rate variability
*LQG*: *Liuzijue qigong*
LSVT: Lee Silverman voice treatment
MCA: Maximal counting ability
MPT: Maximum phonation time
NR: No response
NSOMExs: Non-speech oro-motor exercises
PD: Parkinson’s disease
PR: Partial response
S/Z ratio: Signal–noise ratio
TCM: Traditional Chinese medicine

## References

[1] O'Sullivan SB, Schmitz TJ (2007). Physical rehabilitation 5th ed.

[2] Mozaffarian D, Benjamin EJ, Writing Group M (2016). Heart disease and stroke statistics-2016 update: a report from the American Heart Association. Circulation.

[3] Flowers HL, Silver FL, Fang J (2013). The incidence, co-occurrence, and predictors of dysphagia, dysarthria, and aphasia after first-ever acute ischemic stroke. J Commun Disord.

[4] Simm WA, Roberts PE, Joyce MJ, Clarkson J, Langdon P, Robinson P (2006). Dysarthric speech measures for use in evidence-based speech therapy. Designing accessible technology.

[5] Comrie P, Mackenzie C, McCalls J (2001). The influence of acquired dysarthria on conversational turn-taking. Clin Linguist Phon.

[6] Dickson S, Barbour RS, Brady M (2008). Patients' experiences of disruptions associated with post-stroke dysarthria. Int J Lang Commun Disord.

[7] Schulz GM, Grant MK (2000). Effects of speech therapy and pharmacologic and surgical treatments on voice and speech in Parkinson's disease: a review of the literature. J Commun Disord.

[8] Sapir S, Ramig LO, Fox CM (2011). Intensive voice treatment in Parkinson's disease: Lee Silverman voice treatment. Expert Rev Neurother.

[9] Ramig LO, Fox C, Sapir S (2008). Speech treatment for Parkinson's disease. Expert Rev Neurother.

[10] Fox CM, Ramig LO, Ciucci MR (2006). The science and practice of LSVT/LOUD: neural plasticity-principled approach to treating individuals with Parkinson disease and other neurological disorders. Semin Speech Lang.

[11] Mackenzie C, Muir M, Allen C (2010). Non-speech oro-motor exercise use in acquired dysarthria management: regimes and rationales. Int J Lang Commun Disord.

[12] Mackenzie C, Muir M, Allen C (2014). Non-speech oro-motor exercises in post-stroke dysarthria intervention: a randomized feasibility trial. Int J Lang Commun Disord.

[13] Howard RS, Rudd AG, Wolfe CD (2001). Pathophysiological and clinical aspects of breathing after stroke. Postgrad Med J.

[14] Association CHQ (2004). A brief introduction to health qigong.

[15] Xiao CM, Zhuang YC (2015). Efficacy of Liuzijue qigong in individuals with chronic obstructive pulmonary disease in remission. J Am Geriatr Soc.

[16] Cao LF, Zhao JG (2010). Effects of six-week "Liuzijue" exercise on heart rate variability at different postures. J Biomed Eng Res.

[17] Liu XL, Chen S, Wang Y (2016). Effects of health qigong exercises on relieving symptoms of Parkinson's disease. Evid-Based Complement Alternat Med.

[18] Ranganathan P, Pramesh CS, Aggarwal R (2016). Common pitfalls in statistical analysis: intention-to-treat versus per-protocol analysis. Perspect Clin Res.

[19] Wang CW, Ng SM, Ho RT (2012). The effect of qigong exercise on immunity and infections: a systematic review of controlled trials. Am J Chin Med.

[20] Tsang HW, Cheung L, Lak DC (2002). Qigong as a psychosocial intervention for depressed elderly with chronic physical illnesses. Int J Geriatr Psychiatry.

[21] Mackenzie C (2011). Dysarthria in stroke: a narrative review of its description and the outcome of intervention. Int J Speech Lang Pathol.

[22] Le Huche F, Allali A (1997). From the classification of dysarthria to therapeutic concepts. Rev Laryngol Otol Rhinol (Bord).

[23] Merson RM, Rolnick MI (1998). Speech-language pathology and dysphagia in multiple sclerosis. Phys Med Rehabil Clin N Am.

[24] Morgan AT, Liegeois F (2010). Re-thinking diagnostic classification of the dysarthrias: a developmental perspective. Folia Phoniatr Logop.

[25] Enderby P, Palmer R (2008). Frenchay dysarthria assessment (FDA-2), 2nd ed.

