# Supplementary material for: Liuzijue Qigong vs traditional breathing training for patients with post-stroke dysarthria complicated with abnormal respiratory control: study protocol of a single center randomized controlled trial
Source: Trials. 2018 Jun 26;19:335. doi: 10.1186/s13063-018-2734-0 (PMC6019506; doi:10.1186/s13063-018-2734-0)
Supplement: Supplementary file 1 — SPIRIT 2013 Checklist. (DOCX 55 kb) [file 13063_2018_2734_MOESM1_ESM.docx]

**Additional file 2: Table S1. Primary and secondary objectives as well as their related statistical methods**

|  | Indicator | Efficacy evaluation method | Type of data | Statistical method |
| --- | --- | --- | --- | --- |
| Primary objective | Speech and breathing level of a modified Frenchay Dysarthria Assessment (FDA) | Five levels: a\b\c\d\e. a grade is 1 point, b grade is 2 points, c grade is 3 points, d grade is 4 points, e grade is 5 points, scores were calculated. | Ranked data | 1. The statistical description will be carried out according to the number of cases (%). If it is not specially pointed out, hypothesis testing will use a two-sided test with *P*=0.05 as the statistical cutoff.  2. After the intervention, the value of the score change will be compared within groups. A signed rank sum test will be used. The statistic will be S; The value of the score change will be compared using the Wilcoxon rank sum test within the group and the statistic will be Z.  3. The influence of rehabilitation intervention time, stroke classification, age and gender on the efficacy of intervention method and the relative size of the efficacy will be eliminated, using a stepwise logistic regression model. |
|  |  | Calculate the evaluation of efficacy before and after intervention The criteria is: Excellent: reduce ≥ 2 levels or reduce to level a; Effective: reduced ≥ 1 level; Ineffective: No change in level or increase in severity. | Ranked data | The overall efficacy evaluation will be effective and the efficacy evaluation method adopted. The formula is: Effective rate = (excellent + effective) / total number of cases * 100%. The chi-squared test will be used to compare the difference in efficacy between the two groups. |
| Secondary objective |  |  |  |  |
|  | Modified Frenchay Dysarthria Assessment | The assessment has 8 major items and 28 sub-items, which are classified into 5 levels a, b, c, d, e according to the degree of damage. Dysarthria was quantified by the number of grade a. Subjects were given a score of 1 for each grade a, and totals are calculated. | Measurement data | After the intervention, the change value of the total score will be evaluated. We will use repeated measurement covariance analysis The pre-intervention evaluation value will be covariate and we will determine whether there is a statistically significant difference between groups in the change value of the total score of the observation points after the intervention. |
|  | MPT | After a deep breath, the longest time to keep pronouncing a single vowel/a/ | Measurement data | After the intervention, the MPT change value will be evaluated. We will employ repeated measurement covariance analysis The pre-intervention evaluation value will be covariate. We will determine whether there is a statistically significant difference between groups in the MPT change value of the observation points after the intervention. |
|  | MCA | After a deep breath, the longest time to pronounce 1 in one breath. | Measurement data | After the intervention, the MCA change value will be evaluated. We will use repeated measurement covariance analysis. The pre-intervention evaluation value will be covariate. We will determine whether there is a statistically significant difference between groups in the MCA change value of the observation points after the intervention. |
|  | S/Z | After a deep breath, pronounce /s/ and /z/ separately, and the ratio of the maximum vocalization time of the two is calculated. | Measurement data | After the intervention, the S/Z change value will be evaluated. We will adopt repeated measurement covariance analysis. The pre-intervention evaluation value will be covariate. We will determine whether there is a statistically significant difference between groups in the S/Z change value of the observation points after the intervention. |
|  | Loudness level | The current loudness level of the patient is determined to be at five levels (whispered, soft, conversational, loud, shouting) corresponding to level 1, level 2, level 3, level 4 and level 5, respectively. | Ranked data | 1. The statistical description will be carried out according to the number of cases (%). If it is not specifically pointed out, our hypothesis testing will use a two-sided test with *P*=0.05 as the statistical cutoff.  2. After the intervention, the value of the score change will be compared within the group. A signed rank sum test will be used. The statistic will be S; The value of the score change will be compared using the Wilcoxon rank sum test within the group and the statistic will be Z. |
